# Qingjie Fuzheng granules attenuate cancer cachexia by restoring gut microbiota homeostasis and suppressing IL-6/NF-κB signaling in colorectal adenocarcinoma

**DOI:** 10.1186/s41065-025-00541-1

**Published:** 2025-08-29

**Authors:** Yishun Jin, Lisha Lu, Hangju Hua, Biyin Chen, Wenzheng Fang, Kaimin Lin, Peng Ren, Zhenbo Geng, Ling Wang, Xiaohua Yan, Wujin Chen, Jiumao Lin

**Affiliations:** 1 The third Affiliated people’s Hospital of Fujian University of Traditional Chinese Medicine, No. 363, Guobin Avenue, Shangjie, Minhou, Fuzhou, Fujian Province 350108 China; 2 Department of Traditional Chinese Medicine, Fuzhou University Affiliated Provincial Hospita, Fuzhou, 350001 China; 3 Provincial Hospital Clinical Medical College of Fujian Medical University, Fuzhou, 350001 China; 4 The First Clinical Medical School of Guangzhou University of Chinese Medicine, Guangzhou, Guangdong 510000 China; 5 Oncology Department, The Affiliated People’s Hospital of Fujian University of Traditional Chinese Medicine, Fuzhou, Fujian 350004 China; 6 Department of Pharmacy, Fuzhou University Affiliated Provincial Hospita, Fuzhou, 350001 China; 7 Academy of Integrative Medicine, Fujian University of Traditional Chinese Medicine, Fuzhou, Fujian 350122 China; 8 Fujian Key Laboratory of Integrative Medicine on Geriatrics, Fujian University of Traditional Chinese Medicine, Fuzhou, Fujian 350122 China

**Keywords:** Qingjie Fuzheng granules, Cancer cachexia, Gut microbiota, IL-6/NF-κB, Th17/Treg, Colorectal adenocarcinoma

## Abstract

Cancer cachexia, a debilitating syndrome characterized by muscle wasting and systemic inflammation, remains a major unmet clinical need. Qingjie Fuzheng granules (QFG), a traditional Chinese medicine formulation, have shown promise in cancer therapy, but their role in cachexia management is unclear.

Here, we investigated the anti-cachectic effects of QFG in a murine model of colon adenocarcinoma-induced cachexia. 16S rRNA sequencing revealed gut dysbiosis in cachectic mice, with increased Enterobacteriaceae and decreased Lactobacillus. QFG treatment restored microbial balance, reduced pro-inflammatory cytokines TNF-α, IL-6, and enhanced intestinal barrier integrity by upregulating tight junction proteins ZO-1, Occludin, Calprotectin.

Mechanistically, QFG rebalanced Th17/Treg cell ratios and suppressed IL-6/NF-κB signaling, a key driver of muscle atrophy. Combining QFG with glutamine (Gln) further amplified these effects, suggesting synergistic therapeutic potential.

Our findings demonstrate that QFG ameliorates cancer cachexia through microbiota modulation and IL-6/NF-κB inhibition, providing a novel multi-targeted approach for cachexia treatment.

**Supplementary Information:**

The online version contains supplementary material available at 10.1186/s41065-025-00541-1.

## Introduction

Colorectal adenocarcinoma is one of the most common malignant tumors of the digestive system, ranking third in terms of global incidence and second in mortality among all cancers [1, 2]. In recent years, the incidence of colorectal adenocarcinoma has continued to rise with a trend toward younger age of onset, posing a serious threat to human health [3]. Notably, most patients with advanced colorectal adenocarcinoma develop cancer cachexia (CC) — a multifactorial metabolic syndrome characterized by progressive skeletal muscle wasting (with or without loss of adipose tissue) [2, 4]. Its pathological process cannot be fully reversed by traditional nutritional support, ultimately leading to functional impairment and poor prognosis in patients. It has been reported that 60%-80% of cancer patients develop cachectic manifestations, and approximately 20% of tumor patients die directly from cachexia-related complications [3, 5]. Therefore, elucidating the pathogenesis of cachexia and exploring effective intervention strategies are of great clinical significance for improving the quality of life of patients with advanced cancer.

The pathological mechanism of cachexia is complex, involving multiple links such as inflammatory imbalance, metabolic disorder, and neuroendocrine dysfunction [6]. Among them, the abnormal activation of the gut microbiota-intestinal barrier-immunoinflammation axis is considered a key driving factor [7]. Under healthy conditions, gut microbiota participates in host metabolic homeostasis by maintaining intestinal barrier integrity and regulating immune balance [8, 9]. However, during cancer progression, gut microbiota dysbiosis induced by the tumor microenvironment (e.g., increased abundance of pathogenic bacteria and decreased beneficial bacteria) can disrupt intestinal barrier function, leading to translocation of bacteria and their metabolites, which activates systemic or local inflammatory responses [10, 11]. Studies have confirmed that the levels of proinflammatory cytokines (such as TNF-α, IL-6, and IL-1β) in the serum of cachectic patients are significantly elevated [12–15]. These factors exacerbate skeletal muscle breakdown and fat consumption by activating signaling pathways such as NF-κB, forming a vicious cycle of microbiota dysbiosis-barrier damage-inflammation amplification [15, 16]. In addition, gut microbiota disorder can also affect immune homeostasis by regulating Th17/Treg cell balance — excessive activation of Th17 cells and defective function of Treg cells will further aggravate chronic inflammation and accelerate cachexia progression [17, 18]. Although Western medical intervention strategies targeting inflammatory factors or signaling pathways have been attempted, their clinical application is still limited due to limited efficacy and obvious side effects.

Traditional Chinese medicine (TCM) has the advantage of “holistic regulation and multi-target action” in the management of chronic diseases, providing new ideas for cachexia treatment [19]. Qingjie Fuzheng granules (QFG), a classic TCM formula clinically used as an adjuvant therapy for digestive tract tumors, is composed of Hedyotis diffusa Willd, Scutellaria barbata, Astragalus membranaceus, fried malt, and other herbs, with effects of clearing heat and detoxifying, replenishing qi and strengthening the spleen, and improving appetite [20, 21]. Previous studies have confirmed that QFG can induce apoptosis of colorectal cancer cells by inhibiting signaling pathways such as PI3K/AKT and ERK, suppress tumor migration by regulating the lncRNA ANRIL/let-7a/TGF-β1/Smad axis, and alleviate chemotherapy-induced intestinal mucosal damage and immune function decline [22, 23]. However, whether QFG improves cancer cachexia by regulating gut microbiota and immunoinflammatory networks, its specific mechanism of action, and its synergistic effect with other interventions (such as glutamine, a conditionally essential amino acid involved in intestinal barrier repair) remain unclear .

Based on the above background, this study systematically investigates the ameliorative effect of QFG on colorectal adenocarcinoma-induced cachexia by establishing a murine cachexia model induced by MC-38 colon cancer cells, combined with techniques such as 16S rRNA sequencing, histopathology, and molecular biology. This study aims to clarify the core mechanism by which QFG improves cachexia, providing experimental evidence for its clinical application.

## Experimental materials and methods

### Cell culture

Mouse colon cancer cells MC-38 (iCell, iCell-m032) were purchased from Shanghai Saibaikang Biotechnology Company Limited (ATCC cell bank), and the cells were cultured using DMEM (Gibao, 11965092) complete medium (containing 10% FBS, 1% penicillin-streptomycin) at 37℃ in a 5% CO2 incubator.

### Animal grouping and intervention

C57BL/6 mice 6-month-old adult mice (Spectrum (Beijing) Biotechnology Co., Ltd, License No. SCXK (Beijing) 2019-0010) were divided into 5 groups (*n* = 6), which were classified into (A) Control, (B) Model, (C) Model + QFG, (D) Model + Glutamine (Gln), (E) Model + QFG + Gln, mice in groups B, C, D, and E were inoculated with tumor cells MC-38 cells, 1*105 each, 50ul each, under the plasma membrane of the cecum, and then sutured and sterilized, and the dead animals were replenished in time. Constructed mouse model construction can be completed 1–2 weeks after implantation, continued for 8 weeks after behavioral testing, serum and gastrocnemius muscle collection.

### Fecal specimen collection

Fourteen days after intervention administration, mouse feces are collected with sterile EP tubes. The collected feces should be stored in -80 °C refrigerator in time to avoid repeated freezing and thawing.

### HE staining

Take out the tissue and rinse it with running water for several hours, dehydrate it by 70%, 80%, 90% ethanol solution at all levels, pure alcohol, xylene equal mixture for 15 min, xylene Ⅰ for 15 min, Ⅱ for 15 min (until transparent). Put into the mixture of half of xylene and paraffin for 15 min, and then put into paraffin Ⅰ, paraffin Ⅱ translucent wax for 50–60 min each. paraffin embedding, sectioning. The paraffin slices were baked, then dewaxed and hydrated. Put the sections which have been put into distilled water into hematoxylin aqueous solution for staining for 3 min, hydrochloric acid ethanol differentiation solution for differentiation for 15s, a little water washing, return blue solution for return blue for 15s, rinse with running water, eosin staining for 3 min, rinse with running water, dehydration, transparency, sealing, and microscopic examination and observation.

### qPCR detection

Total RNA in the cells was extracted by Trizon (CW0580S, CWBIO) reagent, mRNA was extracted by RNA Ultra Pure Extraction Kit (CW0581M, CWBIO), the concentration and purity of mRNA were determined by UV-visible spectrophotometer (OD260/OD280), and cDNA was synthesized by RNA Reverse Transcription Kit, and the cDNA was synthesized by Fluorescence The cDNA was synthesized by RNA reverse transcription kit, and fluorescence quantitative PCR was performed using a fluorescence PCR instrument (CFX Connect, Bó Lè Life Medical Products (Shanghai) Co., Ltd.) The reaction steps were as follows: pre-denaturation 95 °C, 10 min; denaturation 95 °C, 10 s; annealing 58 °C, 30 s; extension 72 °C, 30 s; 40 cycles. β-actin was used as an internal reference, and the relative expression of the gene was calculated according to the 2-ΔΔCt method. The primer sequences are shown in the Table 1.

**Table 1:** Primer sequence

Primer names *	Primer Sequence
M.mu IL-1β-F	5’-TGGACCTTCCAGGATGAGGACA-3’
M.mu IL-1β-R	5’-GTTCATCTCGGAGCCTGTAGTG-3’
M.mu IL-6-F	5 ʹ -TACCACTTCACAAGTCGGAGGC-3ʹ
M.mu IL-6-R	5 ʹ -CTGCAAGTGCATCATCGTTGTTC-3ʹ
M.mu TNF-α-F	5 ʹ -CTCTTCTGCCTGCTGCACTTTG-3ʹ
M.mu TNF-α-R	5 ʹ -ATGGGCTACAGGCTTGTCACTC-3ʹ
M.mu occludin-F	5’-TGGCAAGCGATCATACCCAGAG-3’
M.mu occludin-R	5’-CTGCCTGAAGTCATCCACACTC-3’
M.mu claudin-1-F	5’-GGACTGTGGATGTCCTGCGTTT-3’
M.mu claudin-1-R	5’-GCCAATTACCATCAAGGCTCGG-3’
M.mu Calprotectin-F	5’-TGGTGGAAGCACAGTTGGCAAC-3’
M.mu Calprotectin-R	5’-CAGCATCATACACTCCTCAAAGC-3’
M.mu NF-κB-F	5’-GCTGCCAAAGAAGGACACGACA-3’
M.mu NF-κB-R	5’-GGCAGGCTATTGCTCATCACAG-3’
M.mu β-actin-F	5’-CATTGCTGACAGGATGCAGAAGG-3’
M.mu β-actin-R	5’-TGCTGGAAGGTGGACAGTGAGG-3’
M.mu Zonulin-F	5’-GTTGGTACGGTGCCCTGAAAGA-3’
M.mu Zonulin-R	5’-GCTGACAGGTAGGACAGACGAT-3’

### Western blot staining

Tissue samples were taken, and the total protein was extracted with RIPA lysate after the tissue was prepared as tissue suspension by shearing the tissue. 12000r/min high-speed centrifuge was centrifuged at 4℃ for 10 min, and the supernatant was taken, and the total protein was quantified by BCA protein quantification kit (E-BC-K318-M, Elabscience). After denaturation of the protein samples, sodium dodecyl sulfate gel electrophoresis (SDS-PAGE) was performed for 1.5 h, followed by constant flow of the membrane at 300 mA for 1 h. The PVDF membrane (IPVH00010, Millipore) was closed with skimmed milk powder, and the primary antibodies anti-Occludin (CST, Ab#91131), anti-NF-κB (Abcam, ab307840), anti-TNF-α (Cohesion Biosciences, CPA2174), anti-IL-6 (Cohesion Biosciences, CPA4914), anti-IL-1β (Cohesion Biosciences, CPA1586), anti-Zonulin (CST, mAb#13663), anti-Calprotectin (Invitrogen, MAC378), and anti-β-actin (Proteintech, 20536-1-AP) were diluted multiplicatively according to the kit instructions and incubated at 4 °C The next day, the PVDF membrane was incubated with secondary antibodies HRP-Goat anti Rabbit (proteintech, SA00002-1) and HRP-Goat anti Muose (proteintech, SA00002-2) at room temperature for 2 h. The PVDF membrane was wetted with luminescent solution and placed on an ultra-high sensitivity chemiluminescence imaging system (Chemi DocTM XRS+, Bio-Red) for developing and photographing, and the resultant images were analyzed in grayscale using ImageJ.

### Flow cytometry staining

Mouse spleens were taken, clipped, ground completely, and the cell suspension was collected and sieved to obtain spleen single cell suspension. In order to detect the changes in the ratio of Th17 and Treg cells in the spleen, 2 µL each of different indicated antibodies FITC CD4 Antibody (Biolegend,116003) and APC CD25 Antibody (Biolegend,102012) were used in sequence, incubated for 15 min away from light, and the excess antibody was washed and then analyzed by True- Nuclear™ Transcription Factor Buffer Set (Biolegend, 424401) was incubated for 15 min, and the excess antibody was washed, and the membrane and nucleus were fixed with True- Nuclear™ Transcription Factor Buffer Set (Biolegend, 424401) for 40 min, and the membrane and nucleus were broken by adding PE FOXP3 Antibody (Invitrogen, 12-5773-82), Percp IL-17 A Antibody (Biolegend, 506944) antibody 2µL, incubated at room temperature under light for 30 min, washed and resuspended with 500µL PBS, and detected by flow cytometry.

### ELISA staining

Blood was separated from plasma and referred to ELISA for TNF-α (RUIXIN Biotech, Cat.# RX202412M); IGF-1 (RUIXIN Biotech, Cat.# RX202483M); LEP (RUIXIN Biotech, Cat.# RX 202429 M); IgM (RUIXIN Biotech, Cat.# RX202732M); IgG (RUIXIN Biotech, Cat.# RX202736M); IL-1β (RUIXIN Biotech, Cat.# RX 203063 M); IL-18 (RUIXIN Biotech, Cat.# RX 203064 M), expression changes, were detected according to the kit instructions. Absorbance values of each well were measured at the wavelength specified in the kit using an enzyme labeling instrument (SuPerMax3100, Shanghai Flash Spectrum Biotechnology Co., Ltd.).

### 16S sequencing

**Fig. 1:**
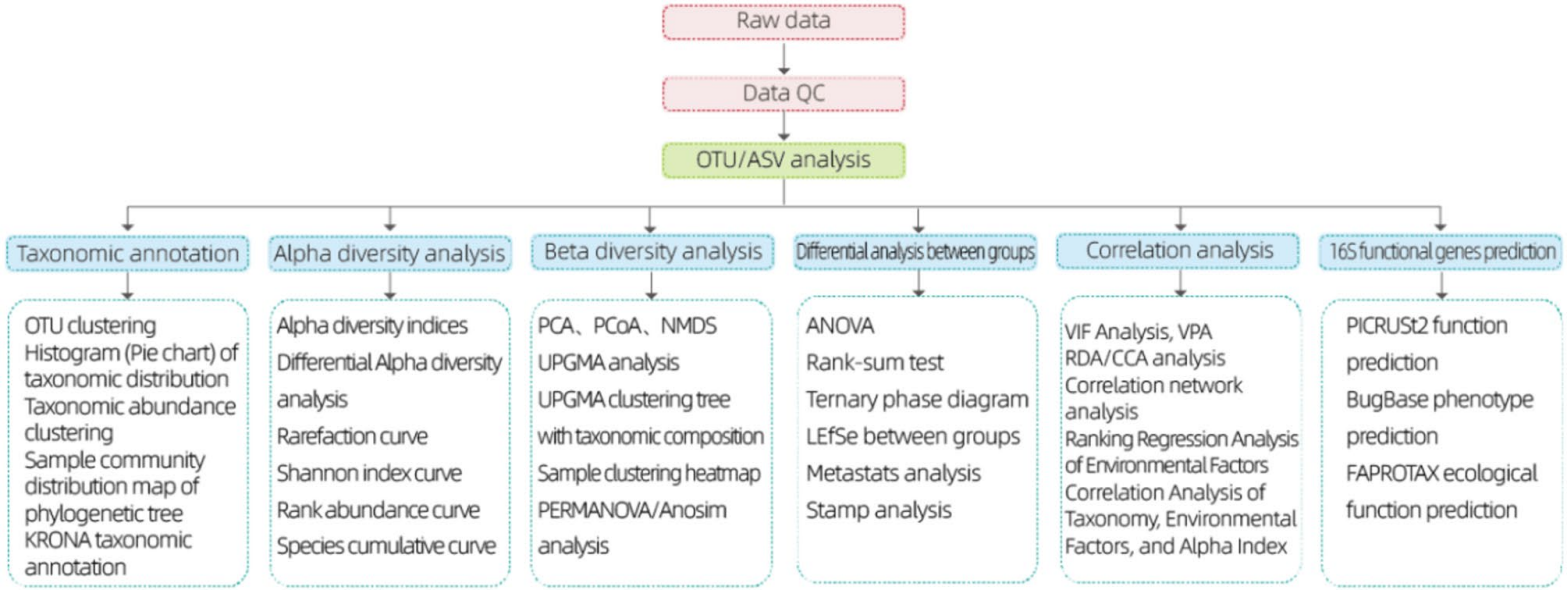
Workflow

The total bacterial DNA in each group of fecal samples was extracted according to E.Z.N.A Mag-Bind Soil DNA Kit, the DNA concentration and purity were detected using Qubit3.0 DNA Detection Kit, and the quality of DNA extraction was detected using 1% agarose gel electrophoresis, and the total DNA of the samples was extracted, and then the primers were obtained according to the design of the conserved region (as shown in Table 2), and a 16S sequencing junction was added to the end of the primers for PCR amplification. After extraction of total DNA, primers were designed according to the conserved region, and the sequencing connector was added to the end of the primers, PCR amplification was carried out, and the products were purified, quantified and homogenized to form sequencing libraries, and the constructed libraries were subjected to library quality control, and those libraries that passed the quality control were sequenced with Illumina NovaSeq 6000. The raw image data files obtained from high-throughput sequencing were converted into original sequenced reads by Base Calling analysis, and the results were stored in FASTQ (abbreviated as fq) file format, which contains the sequence information of the sequenced reads as well as their corresponding sequencing quality information. As shown in Fig. 1, the quality filtering: Firstly, the Raw Reads obtained from sequencing were filtered using Trimmomatic v0.33 software; then, the primer sequences were identified and removed using cutadapt 1.9.1 software, and the Clean Reads without primer sequences were obtained; DADA2 denoising: The dada2 method of QIIME2 2020.6 was used for denoising, and the double-ended sequences were denoised using the dada2 method. method in QIIME2 2020.6 for denoising, double-ended sequence splicing and removing chimeric sequences to get the final valid data (Non-chimeric Reads).As shown in Fig. 1, the information analysis content: division of Feature (OTUs, ASVs), diversity analysis, difference analysis, correlation analysis and functional prediction analysis.

**Table 2:** Primer sequence

F (5’-3’)	*R*(5’-3’)
ACTCCTACGGGAGGCAGCA	GGACTACHVGGGTWTCTAAT

### Statistical analysis

The data in this study were statistically analyzed using GraphPad Prism 8 software. For comparisons involving three or more groups, one-way analysis of variance (ANOVA) was utilized, followed by post hoc pairwise comparisons using the Tukey or Bonferroni corrected multiple comparison tests based on the data distribution. Independent samples t-test was employed for pairwise comparisons between two sets of data. All values are presented as means ± standard deviation (SD), with a significance level set at *P* < 0.05. Additionally, for non-normally distributed data, non-parametric statistical methods such as the Mann-Whitney U test were employed for analysis. For all experiments, this study will ensure a minimum of three replicates for each experiment to guarantee reproducibility and reliability of the results.

## Experimental results

### HE staining examination of colonic histopathological changes after treatment

**Fig. 2:**
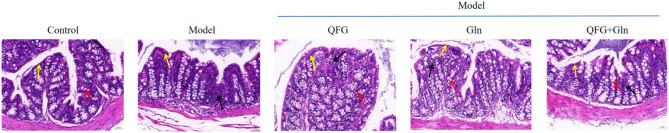
HE was used to detect the histopathological changes of the colon in mice. **Note: The yellow arrows showed mucosal epithelial cells; The red arrows showed cup cells; The black arrows showed inflammatory cells**

In order to investigate the effect of QFG (Qingxie Fuzheng Granule) on the cancerous malignant stroma of mice with colon adenocarcinoma, this study examined the histopathological changes of colon tissues of mice in each group by HE staining. As shown in Fig. 2, the results showed that the overall structure of colon tissue was basically normal, the arrangement of mucosal epithelial cells was more regular, and no mucosal epithelial cells were detached and necrotic, the yellow arrows showed mucosal epithelial cells, and the mucosal layer of the tissue was rich in the number of cup cells, and the red arrows showed cup cells, and the interstitium of the tissue did not see the obvious congestion and expansion of blood vessels, and the tissue did not see the obvious infiltration of inflammation cells; the overall structure of colon tissue in model group was abnormal, and the tissue was more regular, and the arrangement of mucosal epithelial cells was more regular. The overall structure of the colon tissue in the model group was abnormal, the arrangement of mucosal epithelial cells was more regular, and no mucosal epithelial cell detachment necrosis was seen, the yellow arrow showed mucosal epithelial cells, the number of cup-shaped cells in the mucosal layer of the organization was abundant, and a large number of inflammatory cells were seen infiltrating the whole layer of the organization, as shown by the black arrow in the figure; the overall structure of the colon tissue was mildly abnormal after the addition of QFG and Glutamine, and the arrangement of mucosal epithelial cells in the organization was more regular, and no mucosal epithelial cells were seen. The yellow arrow shows the mucosal epithelial cells, the structure of the mucosal layer of the tissue is loose, and a large number of cup-shaped cells can be seen, as shown by the red arrow in the figure, the interstitium of the tissue does not see the blood vessels obviously congested and dilated, and the tissue can be seen in a small number of inflammatory cells infiltration, as shown by the black arrow in the figure. The overall structure of colonic tissue in the combination group returned to normal, the tissue mucosal epithelial cells were arranged more regularly, and no mucosal epithelial cell detachment necrosis was seen, as shown by the yellow arrows for the mucosal epithelial cells, and the structure of the tissue mucosal layer was lax, and a large number of cup-shaped cells could be seen, as shown by the red arrows in the figure, and a small amount of inflammatory cell infiltration could be seen in the tissues, as shown by the black arrows in the figure. The results showed that QFG had a certain restorative effect on the intestinal tissue lesions at the place caused by malignant fluid.

### Intestinal microbiota sequencing bioinformatics analysis

#### Species classification and abundance analysis of samples

**Fig. 3:**
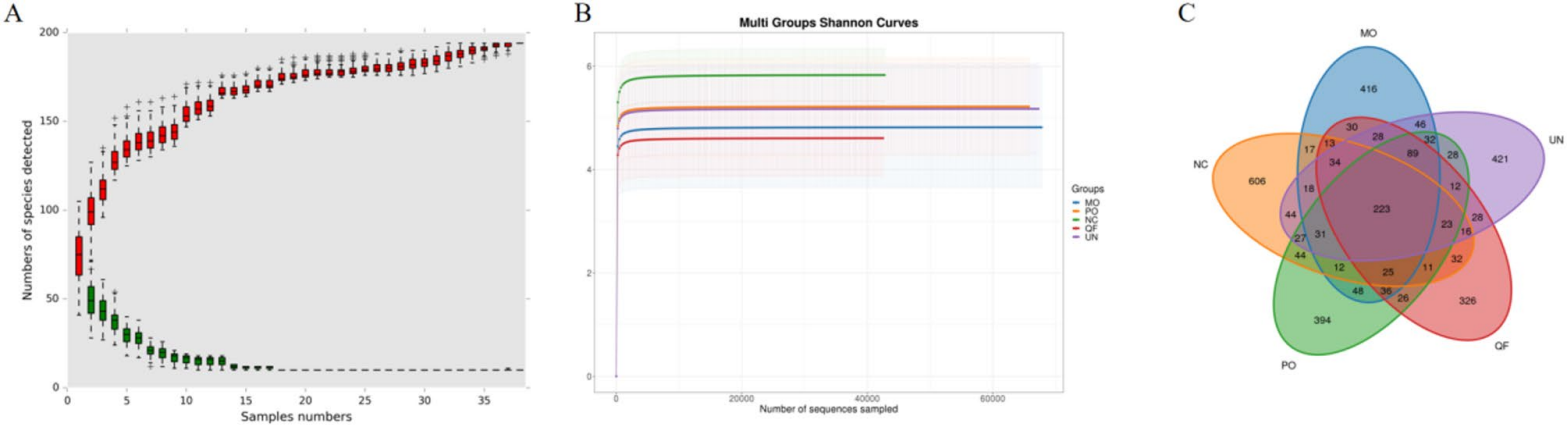
16S was used to detect the classification and abundance of intestinal microbes in each group. Figure **A**. Genus level species accumulation curve. X-axis represents sample amount; Y-axis represents species number after sampling; red boxes form accumulation curve; green boxes form shared amount curve. Figure **B**. Shannon index rarefaction curve. X-axis: Counts of randomly sampled sequences; Y-axis: Corresponding Shannon index. The number of detected species will be growing with more sequencing data is included. Once the counts of species reach saturation, there will not be more species detected with more sequencing data sampled. Figure **C**. Venn Diagram on features. In venn diagram (2–5 samples), each circle represents a sample or group. The overlaps indicates common features among samples and the non-overlapped area indicates unique feature in each sample. Note: NC (control group); MO (model group); QF (Qingjie Fuzheng granules); PO (glutamine-treated group); UN (co-treated group); *n* = 8

The sampling size can be judged by the species accumulation curve: a single red box reflects the total number of species contained in the sample, and the total red box shape forms a cumulative curve, reflecting the rate of emergence of new species under continuous sampling, within a certain range, with the increase of sample size, if the curve shows a sharp increase, it means that a large number of new species have been discovered in the community, and when the curve tends to flatten, it means that the species in this environment will not increase significantly with the increase of sample size; The individual green boxes reflect the number of species common to the sample; Within a certain range, with the increase of sample size, if the curve decreases, it means that the newly discovered common species in the sample are gradually decreasing, and when the curve tends to be flat, it means that the common species in the environment tend to be saturated. The species and function accumulation curve can be used as a judgment on whether the sample size is sufficient, and the sharp increase in the curve indicates that the sample size is insufficient and the sample size needs to be increased. Conversely, it indicates that the sampling is sufficient for data analysis. As shown in Fig. 3A, the curve showed a trend of first rising and then flattening, i.e., the sample size was relatively sufficient and the empirical analysis could be continued. The Shannon diversity index dilution curve was plotted to reflect the microbial diversity of each sample at different sequencing quantities. The larger the Shannon index, the more species and species are richer, indicating that the vast majority of microbial species information has been included in the sample. As shown in Fig. 3B, the species richness of each group was divided into control group > glutamine treatment group > combined treatment group > model group > Qingjie Fuzheng granule treatment group. As shown in Fig. 3C, there were 606 unique OTUs in the control group, 416 in the model group, 326 unique OTUs in the Qingjie Fuzheng granule treatment group, 394 OTUs in the glutamine treatment group, 421 in the combined group, and 223 OTUs in the five groups.

#### Alpha diversity analysis of microbiota differences

**Fig. 4:**
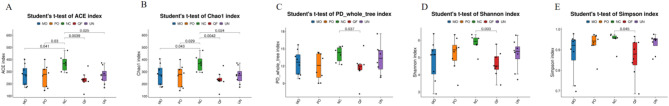
Boxplot of alpha diversity indices. Note: X-axis: Group name; Y-axis: Alpha diversity indices. In the box plot, the lines divide the group into quartiles. Upper and lower quartile line (edges of the box): inter-quartile range (IQR, Q1 to Q3). Middle quartile line: Median; Upper and lower whisker: values outside the middle 50% (Q1-1.5*IQR to Q3 + 1.5*IQR). The dots outside whisker region are regarded as outliers. The value indicates *P*-value calculated by T-test (If the *P* value > 0.05, the *P* value is not displayed by default), *n* = 8

Alpha diversity reflects the species richness and diversity of a single sample, and there are multiple measures: Chao1, Ace, Shannon, and Simpson. The Chao1 and Ace indices measure species richness, i.e., the number of species. The Shannon and Simpson index is used to measure species diversity and is affected by species richness and community evenness in the sample community. In the case of the same species richness, the greater the uniformity of each species in the community, the greater the diversity of the community, and the larger the Shannon index and Simpson index values, the higher the species diversity of the sample. As shown in Fig. 4, the species richness of each group was divided into control group > glutamine treatment group > combined treatment group > model group > Qingjie Fuzheng granule treatment group. The results showed that the richness and species diversity of intestinal microbiota in cancer cachexia mice decreased, and the combined drug intervention could improve the material diversity and richness of intestinal microbiota, but the effect of QFG treatment alone on the diversity and richness of intestinal microbiota was not significant enough.

#### Beta diversity analysis based on OTU

**Fig. 5:**
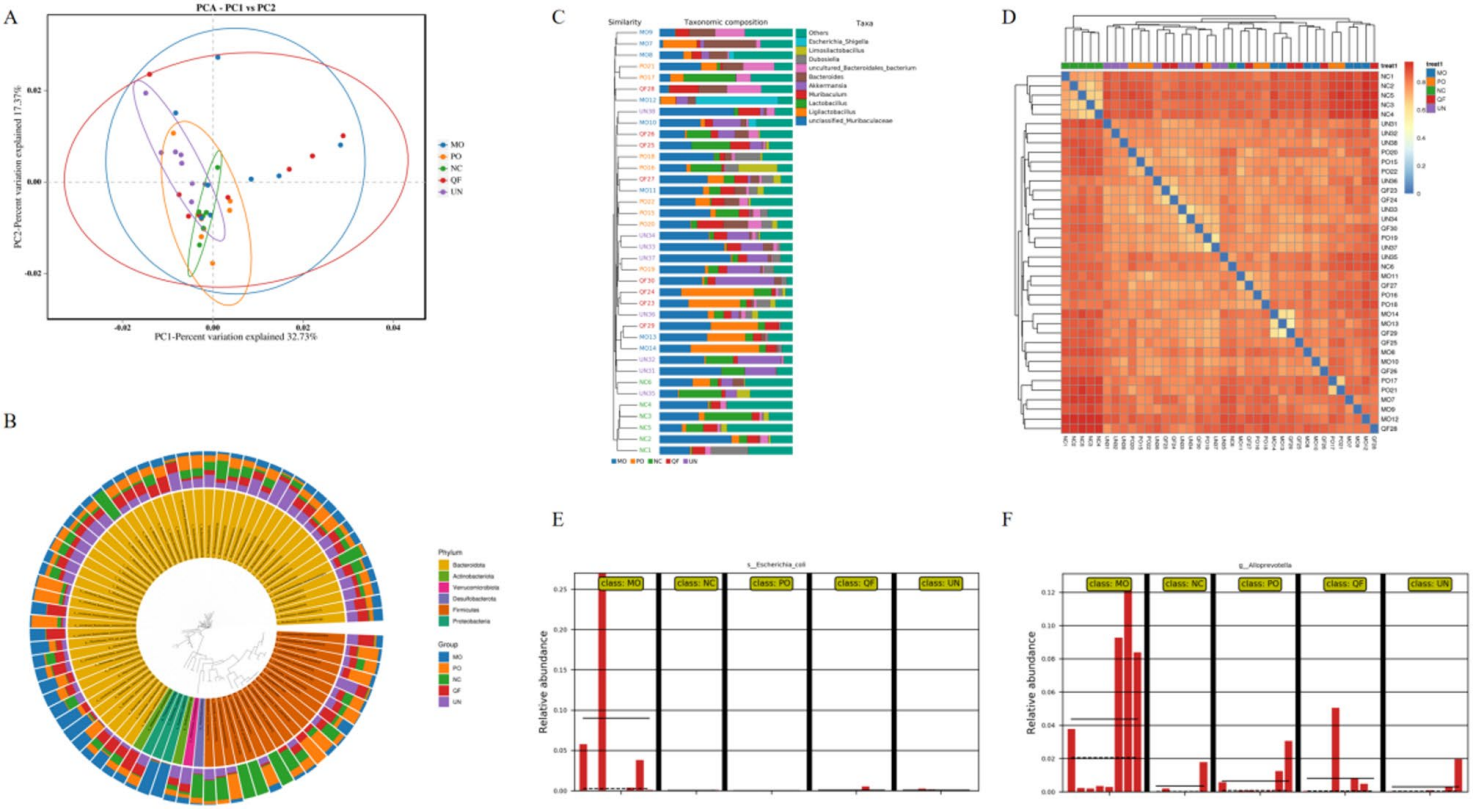
Beta diversity analysis and differential analysis between groups. Figure **A**. PCA analysis graph. Each dot represents a sample. The samples were coloured based on grouping information if applicable. The confidence ellipse defines the region that contains 95% of all samples that can be drawn from underlying Gaussian distribution. X-axis: First principal component. The value in percentage indicates contribution of PC1 in variability. Y-axis: Second principal component. The value in percentage indicates contribution of PC2 in variability. Figure **B**. Combining drawing of clustering tree and histogram. The figure legend in the bottom left represents the color of each group in which the cluster tree samples are grouped. The legend in the top right represents the top 10 species in abundance. The rest are classified as Others. Unannotated species are classified as Unclassified. Figure **C**. Sample distance clustering heat map. The grouping information, if applicable, was shown on the heatmap as coloured blocks. Figure **D**. Sample community distribution map of Phylogenetic tree. Colour corresponding phylum names were shown in the figure legend. Figure **E/F**. Marker intergroup abundance histogram. LEfSe is able to display distribution of relative abundance in different groups (in class). The solid and dash line represents the average and median of relative abundance in each group. Note: NC (control group); MO (model group); QF (Qingjie Fuzheng granules); PO (glutamine-treated group); UN (co-treated group); *n* = 8

Principal Component Analysis (PCA) is a technique for analysing and simplifying data sets by decomposing variances to reflect differences between multiple sets of data on a two-dimensional coordinate plot. PCA uses variance decomposition to reflect the differences of multiple sets of data on the two-dimensional coordinate graph, and the coordinate axes are taken as the two eigenvalues that can reflect the variance to the maximum. The closer the two samples are, the more similar the composition of the two samples is. As shown in Fig. 5A, PC1 and PC2 contributed 32.73% and 17.3% respectively to distinguish the differences between samples, which can better improve the distribution of communities between regional groups. The Top80 donut charts at the OTU/ASV taxonomic level were selected as species evolution trees, and the species names of the same colour (showing the accurately annotated taxonomic species names and corresponding Feature IDs) represented the same phylum. The characteristic sequences of degree proportions were compared with multiple sequences and phylogenetic trees were constructed by QIIME command line, and then the data of phylogenetic trees and taxonomic abundances were combined. As shown in Fig. 5B, the dominant phyla were Firmicutes and Bacteroidota. Beta diversity data are visualised graphically, the analysis plots at the phylum and order levels are shown in Figures A-F, the matrix heat map is the darker the colour, the higher the abundance of species, and vice versa, the lighter the colour, the lower the abundance of species, and the combination of tree and histogram directly observes the proportion of species through the length of the bar. Compared with the normal group, the dominant phyla of the five groups were Bacteroidetes and Firmicutes, followed by Proteobacteria, and the levels of Enterobacteriaceae and Parabacteroides goldsteinii increased in the model group. The results indicated that the combination of drug intervention could increase the number of bacteroides in the intestinal tract of mice, and reduce the phylum Firmicutes and Proteobacteria, which tended to normal levels. As can be seen from Fig. 5C-F, the blank group still has the most dark colours and the most abundant species at the order level, while the model group has the lightest colour and the least species. Among them, the average abundance was the highest in the order Bacteroides, followed by Clostridium, the lowest abundance in the model group, and similar in the other groups, and the highest abundance in the model group of Enterobacteriaceae and Bacterium, indicating that the abundance of Bacteroides in the intestinal tract and the abundance of Enterobacteriaceae and Bacterium were decreased after the intervention of QFG and glutamine. These microbiota are closely related to intestinal inflammation and the occurrence of intestinal barrier lesions, so it is speculated that the regulation of intestinal flora by cleaning and correcting glutamine plays a therapeutic role in regulating the occurrence of cancer cachexia in mice. However, further research is needed on its specific mode of action and mechanism.

### ELISA to detect changes in immune-related factors after drug treatment

**Fig. 6:**
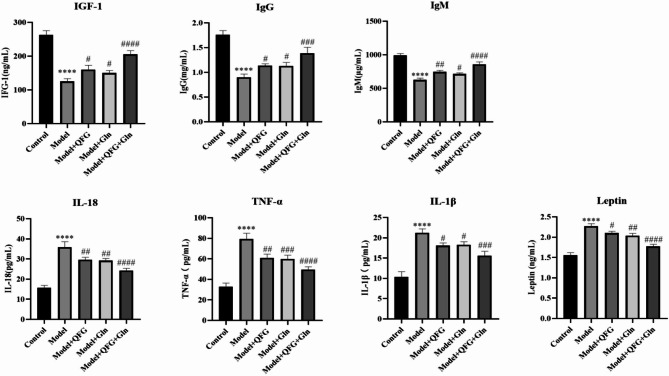
Changes in immune-related factors detected by ELISA. Note: Changes in expression of IgG, IgM, IGF1, TNF-a, IL-18, IL-1β, Leptin in plasma of mice detected by ELISA,^****^P < 0.0001 *VS* Control; ^*#*^*P* < 0.05 ,^*##*^*P* < 0.01,^*###*^*P* < 0.001,^*####*^*P* < 0.0001 *VS* Model, *n* = 3

In order to investigate the effect of QFG on the immune environment factors of cancerous malignant mice, this study detected the changes of immune-related factors in plasma by ELISA. The results are shown in Fig. 6, compared with the control group, the expression of IgG, IgM, IGF1, decreased in the model group; the expression increased after adding drug treatment. Compared with the control group, the expression of TNF-a, IL-18, IL-1β, and Leptin increased significantly in the model group; the expression decreased after drug intervention; the most significant decrease was observed in the combination group. The results showed that the inflammation level decreased after adding drug intervention, and the effect of combined drug treatment was more significant.

### Flow assay to detect changes in the ratio of Th17 and Treg cells after drug treatment

**Fig. 7:**
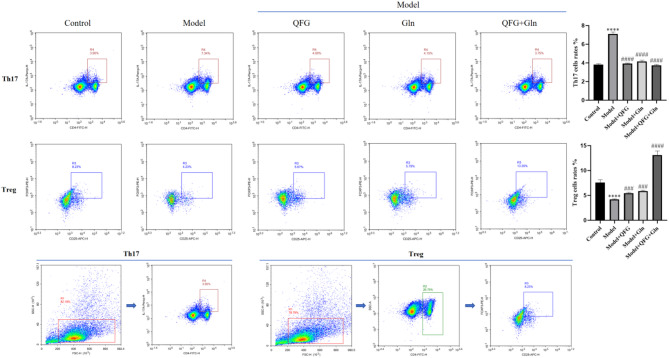
Change in the ratio of Th17 to Treg cells detected by flow cytometry. Note: Flow cytometry detection of changes in the ratio of Th17 to Treg cells in the spleens of mice.^****^P < 0.0001 *VS* Control; ^*###*^*P* < 0.001,^*####*^*P* < 0.0001 *VS* Model, *n* = 3

In order to investigate the effect of QFG on the immune system of cancerous malignant mice, this study detected the changes of Th17 and Treg cells in the spleens of mice by flow assay. The results are shown in Fig. 7, compared with the control group, the proportion of Th17 cells increased and the proportion of Treg cells decreased in the model group; the proportion of Th17 cells decreased and the proportion of Treg cells increased after drug intervention. The results showed that the addition of drug intervention that promotes the rise of the proportion of Treg cells, inhibits the differentiation of Th17 cells, and enhances the immune ability of mice.

### WB detection of changes in inflammation-related pathways

**Fig. 8:**
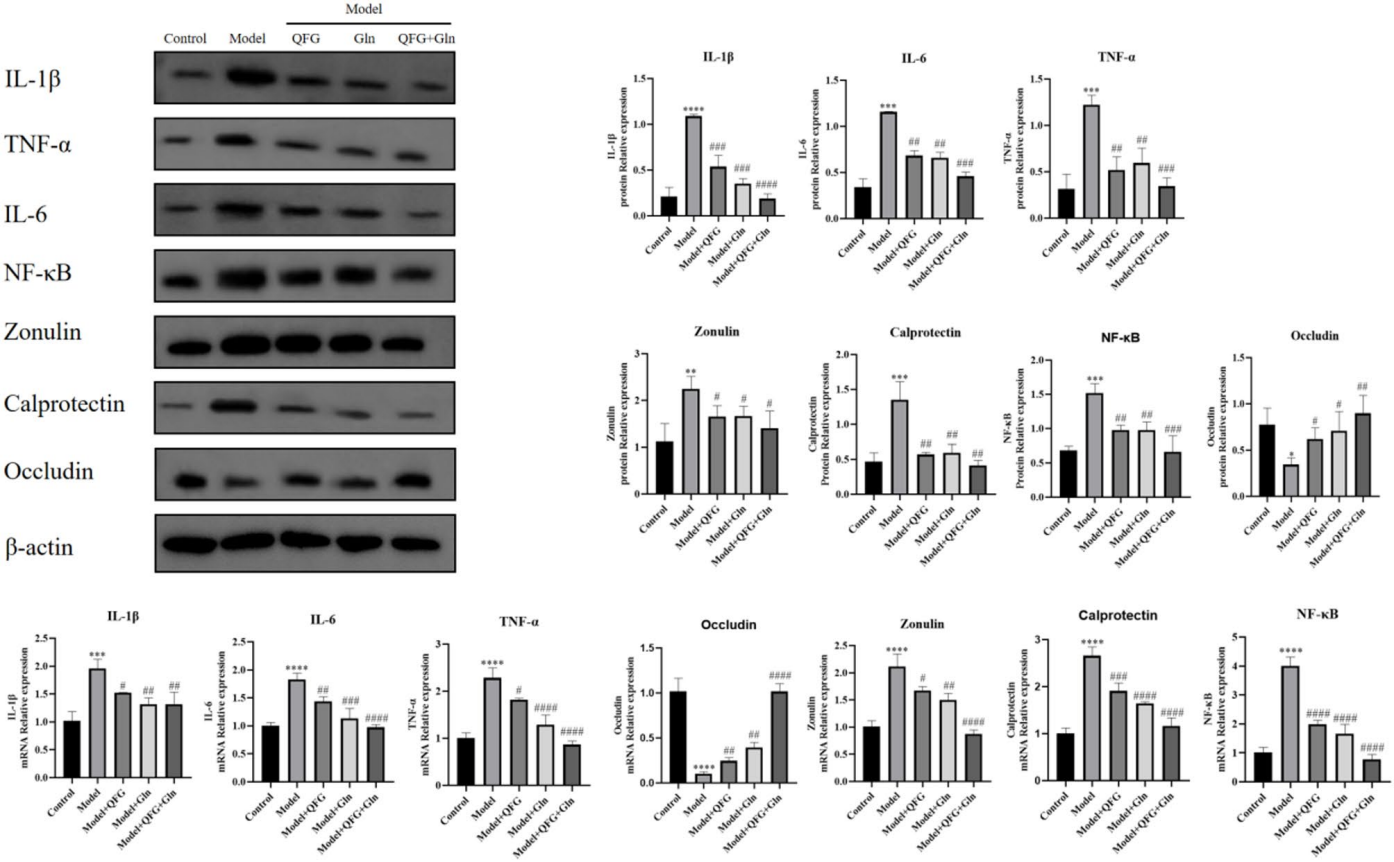
WB detection of relevant protein expression changes. Note: WB detection of changes in protein expression of IL-1β, IL-6, TNF-α, Zonulin, Calprotectin, NF-κB, occludin in mouse colon tissue.^*^P < 0.05, ^**^P < 0.01,^***^P < 0.001,^****^P < 0.0001 *VS* Control; ^*#*^*P* < 0.05 ,^*##*^*P* < 0.01,^*###*^*P* < 0.001,^*####*^*P* < 0.0001 *VS* Model, *n* = 3

In order to study the mechanism of action of drug treatment for cancer cachexia, this study detected changes in protein expression of related pathways by WB. The results, as shown in Fig. 8, showed that compared with the control group, the expression of IL-1β, IL-6, TNF-α, Zonulin, Calprotectin and NF-κB was increased, and the expression of occludin was decreased; the expression of IL-1β IL-6, TNF-α, Zonulin, Calprotectin and NF-κB was decreased after the addition of drug intervention, and the expression of occludin expression increased. The results suggest that the pathway of action of QFG for the treatment of cancer cachexia may work through the IL-6/NF-κB pathway.

## Discussion

Cancer cachexia (CC) remains a critical unmet clinical challenge in advanced colorectal adenocarcinoma, characterized by intractable skeletal muscle wasting, systemic inflammation, and poor response to conventional therapies [24, 25]. This study systematically investigated the therapeutic effect of Qingjie Fuzheng granules (QFG) on colorectal adenocarcinoma-induced cachexia and its underlying mechanisms, providing novel insights into the role of traditional Chinese medicine (TCM) in regulating the gut microbiota-immune-inflammatory axis [26–29]. Here, we integrate our key findings with existing literature to elaborate on the scientific significance and potential clinical implications of this work.

Our results demonstrate that QFG alleviates CC through a multi-target regulatory network involving gut microbiota modulation, intestinal barrier repair, immune homeostasis restoration, and inhibition of pro-inflammatory signaling. This is supported by multiple lines of evidence: first, 16S rRNA sequencing revealed that QFG reversed gut dysbiosis in cachectic mice, characterized by reduced abundance of Enterobacteriaceae and Parabacteroides goldsteinii—taxa previously linked to intestinal inflammation and barrier disruption—and restored levels of lactic acid bacteria, which are critical for maintaining intestinal homeostasis [30, 31]. Second, histopathological and molecular analyses confirmed that QFG repaired colon mucosal damage and upregulated the expression of tight junction proteins ZO-1, Occludin, Calprotectin, which are essential for preventing bacterial translocation and maintaining barrier integrity. Third, flow cytometry results showed that QFG normalized the Th17/Treg balance by reducing pro-inflammatory Th17 cells and increasing regulatory Treg cells, thereby mitigating chronic immune activation—a key driver of cachexia progression. Finally, Western blot and ELISA analyses consistently demonstrated that QFG downregulated pro-inflammatory cytokines TNF-α, IL-6, IL-1β and inhibited the NF-κB pathway, breaking the inflammation-muscle wasting cycle .

The gut microbiota has emerged as a pivotal regulator of CC pathogenesis, with dysbiosis contributing to barrier dysfunction, systemic inflammation, and metabolic derangement [4, 32]. Our study highlights that QFG exerts a corrective effect on the cachectic gut microbiota, which aligns with growing evidence that TCM formulas can modulate microbial ecology to treat disease. Specifically, the reduction in Enterobacteriaceae (a family encompassing pathogenic species like E. coli) following QFG intervention is noteworthy, as overgrowth of this taxa is associated with increased intestinal permeability and endotoxemia in cancer models. Conversely, the restoration of lactic acid bacteria may contribute to barrier repair through metabolite-mediated enhancement of tight junction integrity, providing a plausible link between QFG-induced microbiota changes and improved intestinal function. This finding extends previous reports on QFG’s anti-tumor effects by identifying gut microbiota modulation as a novel mechanism underlying its therapeutic benefit in CC.

The intestinal barrier serves as a physical and immunological barrier against microbial translocation, and its disruption is a hallmark of CC [33, 34]. Our results show that QFG upregulates tight junction proteins (Occludin, ZO-1) at both mRNA and protein levels, which is consistent with prior observations that TCM can protect mucosal integrity. Notably, this effect was potentiated when QFG was combined with glutamine (Gln), a conditionally essential amino acid known to support intestinal epithelial cell proliferation and barrier function [31]. The synergistic improvement in barrier integrity in the QFG + Gln group may explain the more pronounced reduction in pro-inflammatory cytokines and microbial dysbiosis observed in this cohort, as intact barriers limit the release of microbial-associated molecular patterns (MAMPs) that drive NF-κB activation.

Furthermore, the restoration of Th17/Treg balance by QFG—particularly in combination with Gln—adds another layer to its immunomodulatory role [35, 36]. Th17 cells promote chronic inflammation through IL-17 secretion, while Treg cells suppress excessive immune responses; their imbalance is implicated in cachexia-related inflammation [36, 37]. Our finding that QFG shifts this balance toward immune homeostasis aligns with reports that microbiota-derived metabolites (e.g., short-chain fatty acids) can regulate T cell differentiation, suggesting that QFG may act partially through microbial metabolites to modulate adaptive immunity.

Chronic activation of the TNF-α/NF-κB pathway is a central driver of muscle wasting and systemic inflammation in CC [38, 39]. Our data demonstrate that QFG downregulates TNF-α, IL-6, and NF-κB expression in colon tissue, which is consistent with previous studies showing that QFG inhibits pro-inflammatory signaling in colorectal cancer. Mechanistically, the reduction in pro-inflammatory cytokines may stem from both direct inhibition of pathway activation and indirect effects via microbiota/barrier improvement, as reduced bacterial translocation would limit ligand-dependent NF-κB activation [40, 41]. The synergistic effect of QFG + Gln on NF-κB inhibition further supports a multi-targeted approach, as Gln has been shown to suppress NF-κB in models of intestinal inflammation, complementing QFG’s action. This dual regulation of the TNF-α/NF-κB pathway—via both direct and microbiota/barrier-mediated mechanisms—highlights QFG’s advantage as a multi-component therapy for CC, where single-target agents have historically failed.

This study is the first to demonstrate that QFG ameliorates colorectal adenocarcinoma-induced cachexia through a integrated mechanism involving gut microbiota modulation, barrier repair, immune balance, and inflammation suppression. Compared to Western medical strategies (e.g., cytokine-targeting antibodies) that often have limited efficacy or adverse effects, QFG offers a holistic regulation advantage, aligning with TCM principles of treating complex syndromes through multi-target modulation [41–43]. The observed synergism with Gln further supports combination therapy as a promising strategy, as Gln is clinically accessible and well-tolerated, making this approach translationally feasible.

Our findings also expand the understanding of QFG’s therapeutic spectrum beyond anti-tumor and chemo-protective effects to include cachexia amelioration, reinforcing its potential as a multi-functional adjuvant in colorectal cancer management. By linking QFG’s effects to the gut microbiota-immune-inflammatory axis, this work provides a scientific rationale for its clinical use in improving quality of life and survival in advanced cancer patients.

Despite these insights, several limitations should be acknowledged. First, this study was conducted in a murine model, and the translatability of findings to human CC requires validation in clinical cohorts. Second, while we identified key microbiota taxa and pathways, the specific microbial metabolites mediating QFG’s effects (e.g., short-chain fatty acids, bile acids) remain to be characterized. Third, the molecular mechanisms underlying QFG-Gln synergism—particularly at the level of cellular signaling crosstalk—warrant further investigation [43]. Future studies should employ germ-free or gnotobiotic mouse models to confirm causality between microbiota changes and cachexia amelioration, and conduct metabolomic analyses to identify functional metabolites. For QFG, multi-omics (metagenomics, metabolomics) can dissect microbiota-host signaling axes, while AI algorithms may optimize QFG-Gln dosing regimens based on patient-specific microbiota profiles. Additionally, clinical trials are needed to evaluate QFG’s efficacy in human CC, with a focus on patient-reported outcomes and survival.

In conclusion, QFG alleviates colorectal adenocarcinoma cachexia through a distinctive multi-target network, with synergistic potential with glutamine. Its mechanisms align with emerging insights into microbiota-immune crosstalk in CC, and integration with modern technologies promises to advance its clinical translation. These findings support QFG as a promising adjuvant for improving cachexia management in advanced colorectal cancer.

## Supplementary Information

Below is the link to the electronic supplementary material.

Supplementary Material 1

## Data Availability

No datasets were generated or analysed during the current study.
