# Correction: Qingjie Fuzheng granules attenuate cancer cachexia by restoring gut microbiota homeostasis and suppressing IL-6/NF-κB signaling in colorectal adenocarcinoma

**DOI:** 10.1186/s41065-025-00577-3

**Published:** 2025-09-17

**Authors:** Yishun Jin, Lisha Lu, Hangju Hua, Biyin Chen, Wenzheng Fang, Kaimin Lin, Peng Ren, Zhenbo Geng, Ling Wang, Xiaohua Yan, Wujin Chen, Jiumao Lin

**Affiliations:** 1 The third Affiliated people’s Hospital of Fujian University of Traditional Chinese Medicine, No. 363, Guobin Avenue, Shangjie, Minhou, Fuzhou, 350108 Fujian Province China; 2 Department of Traditional Chinese Medicine, Fuzhou University Affiliated Provincial Hospita, Fuzhou, 350001 China; 3 Provincial Hospital Clinical Medical College of Fujian Medical University, Fuzhou, 350001 China; 4 The First Clinical Medical School of Guangzhou University of Chinese Medicine, Guangzhou, 510000 Guangdong China; 5 Oncology Department, The Affiliated People’s Hospital of Fujian University of Traditional Chinese Medicine, Fuzhou, 350004 Fujian China; 6 Department of Pharmacy, Fuzhou University Affiliated Provincial Hospital, Fuzhou, 350001 China; 7 Academy of Integrative Medicine, Fujian University of Traditional Chinese Medicine, Fuzhou, 350122 Fujian China; 8 Fujian Key Laboratory of Integrative Medicine on Geriatrics, Fujian University of Traditional Chinese Medicine, Fuzhou, 350122 Fujian China

**Correction: Hereditas 162**, **178 (2025)**

10.1186/s41065-025-00541-1

Following publication of the original article [1], the authors identified an inaccuracy in the translation of the Chinese herbal medicine “清解扶正颗粒”. Throughout the text, the full name was incorrectly written as **“Qingxie Fuzheng granules”**. The correct translation for the full name should be **“Qingjie Fuzheng granules”**.

Below are the exact locations where the full name appears:

**Table:** 

Page	Location
1	Article title
1	Abstract, line 2
2	Paragraph 1, line 5
2	Paragraph 1, line 7
2	Paragraph 2, line 1
3	Paragraph 1, line 1
5	Figure 2 caption
6	Figure 3 caption
7	Figure 4 caption
10–11	Discussion, Paragraph 1, line 1 and 3

The original article has been updated.
