# Supplementary figures and images for: Qingjie Fuzheng granules attenuate cancer cachexia by restoring gut microbiota homeostasis and suppressing IL-6/NF-κB signaling in colorectal adenocarcinoma
Source: Hereditas. 2025 Aug 29;162:178. doi: 10.1186/s41065-025-00541-1 (PMC12398093; doi:10.1186/s41065-025-00541-1)

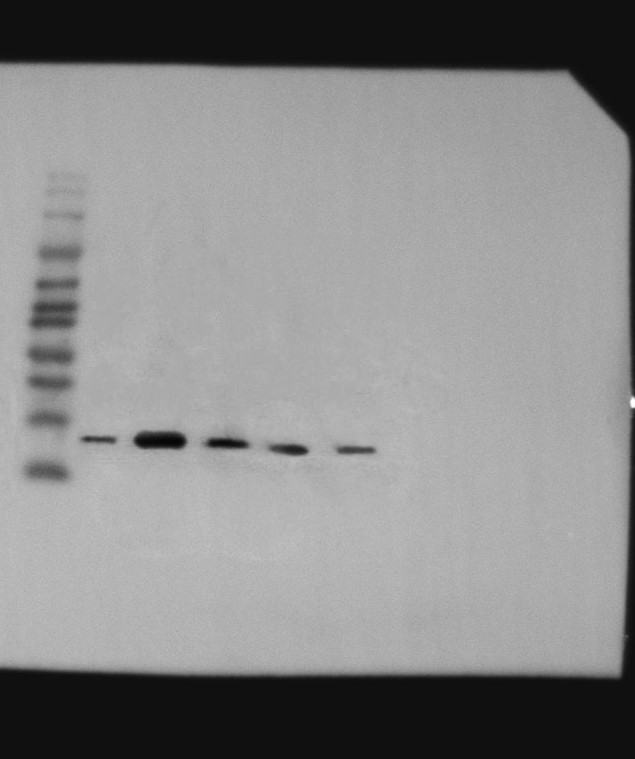

Supplement: Supplementary file 1 — Supplementary Material 1 [file 41065_2025_541_MOESM1_ESM.zip › figure-8WB origical figures/Calprotectin-1.jpg]

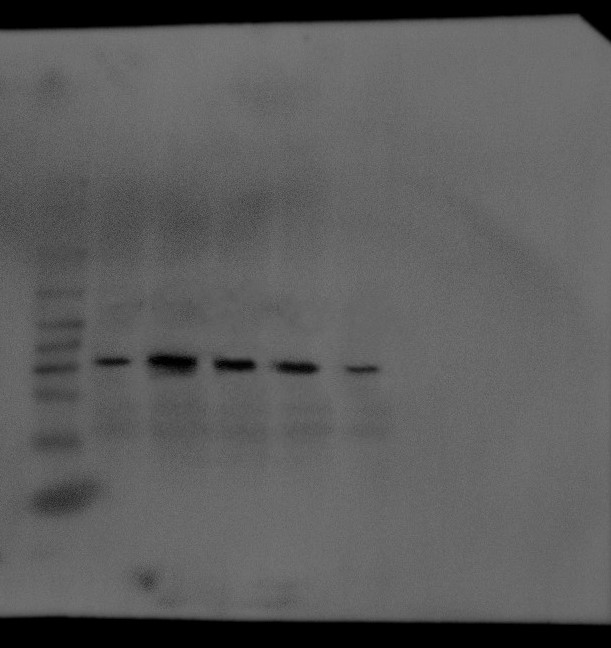

Supplement: Supplementary file 1 — Supplementary Material 1 [file 41065_2025_541_MOESM1_ESM.zip › figure-8WB origical figures/IL-6-1.jpg]

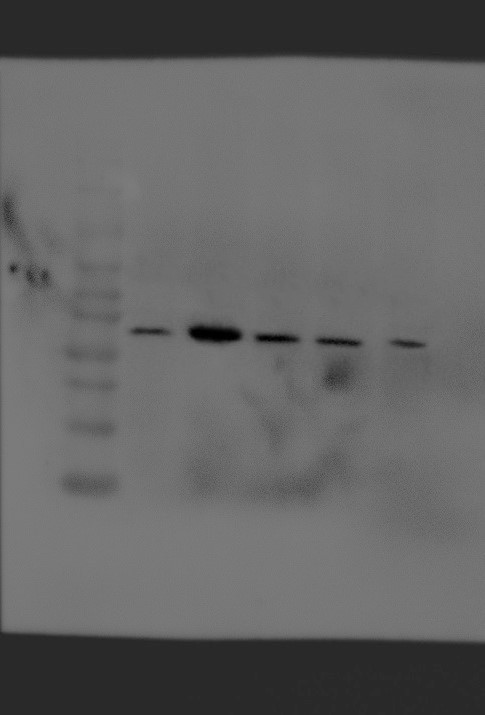

Supplement: Supplementary file 1 — Supplementary Material 1 [file 41065_2025_541_MOESM1_ESM.zip › figure-8WB origical figures/IL-a┬-2.jpg]

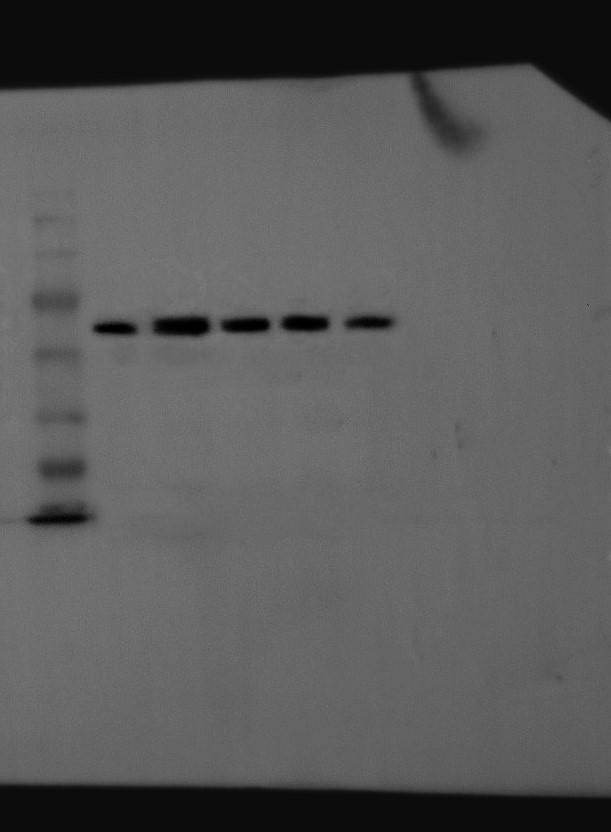

Supplement: Supplementary file 1 — Supplementary Material 1 [file 41065_2025_541_MOESM1_ESM.zip › figure-8WB origical figures/NF-KB-1.jpg]

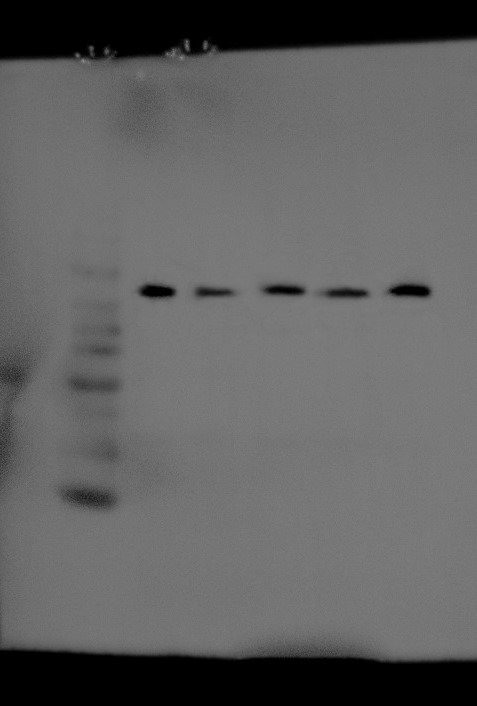

Supplement: Supplementary file 1 — Supplementary Material 1 [file 41065_2025_541_MOESM1_ESM.zip › figure-8WB origical figures/occlvding-1.jpg]

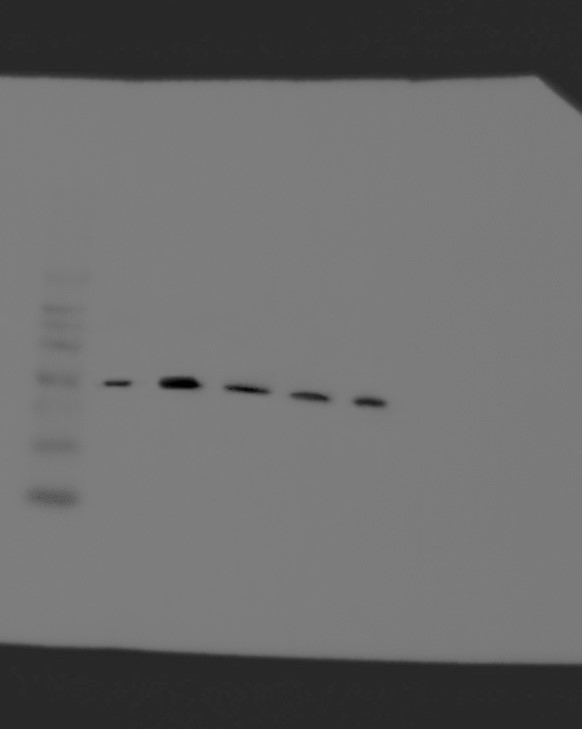

Supplement: Supplementary file 1 — Supplementary Material 1 [file 41065_2025_541_MOESM1_ESM.zip › figure-8WB origical figures/TNF-a-2.jpg]

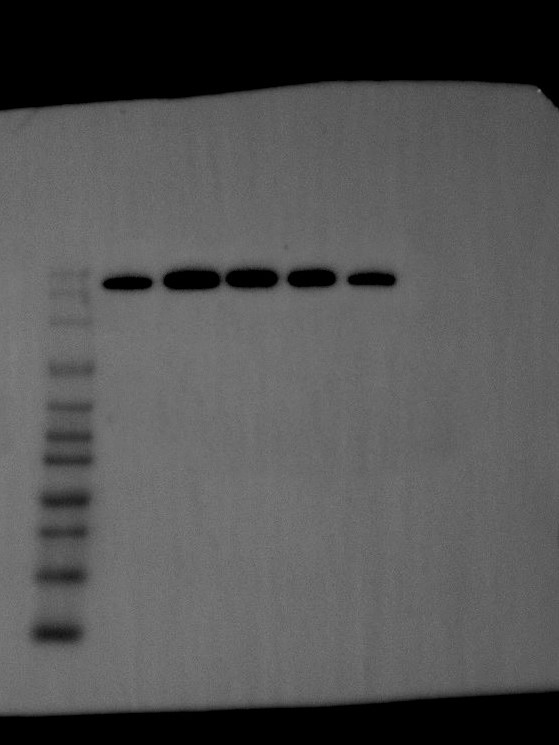

Supplement: Supplementary file 1 — Supplementary Material 1 [file 41065_2025_541_MOESM1_ESM.zip › figure-8WB origical figures/zonulin-1.jpg]

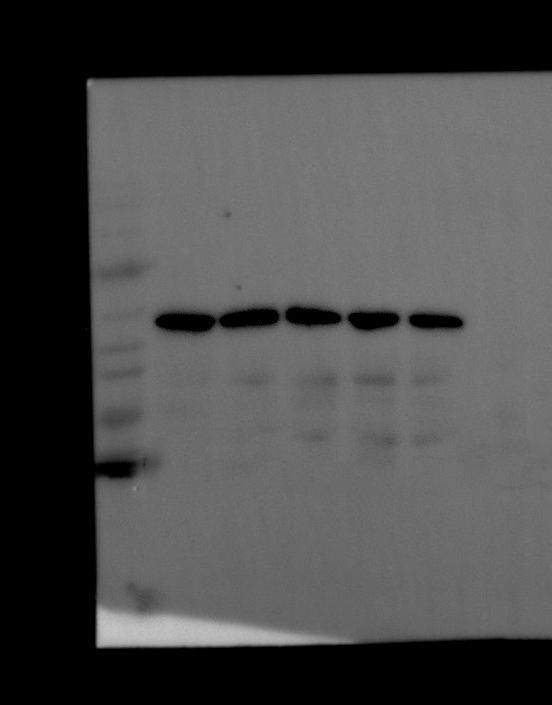

Supplement: Supplementary file 1 — Supplementary Material 1 [file 41065_2025_541_MOESM1_ESM.zip › figure-8WB origical figures/a┬-actin-2(1).jpg]
